# A sustainable, trainee-led CASC course model: addressing IMG attainment gaps through simulation-based education

**DOI:** 10.1192/bjb.2025.10202

**Published:** 2026-04

**Authors:** Chirag Shroff, Zara Ahmed, Indira Vinjamuri, Fiona Douglas, Nachiket Shankar, Ranjan Baruah

**Affiliations:** ST6 Higher Psychiatry Resident, General Adult Psychiatry, Mersey Care NHS Foundation Trust, Prescot, UK; Director of Medical Education, Mersey Care NHS Foundation Trust, Prescot, UK; ST6 Higher Psychiatry Resident, General Adult Psychiatry, Cambridge and Peterborough NHS Foundation Trust, Cambridge, UK; Professor, Department of Anatomy and Medical Education, St John’s Medical College Hospital, Bangalore, India; Consultant Psychiatrist, Mersey Care NHS Foundation Trust, Prescot, UK

The Clinical Assessment of Skills and Competencies (CASC) is the final component of the MRCPsych examinations, assessing trainees’ clinical and communication skills through simulated situations. During the COVID-19 pandemic, the examination was delivered online. In 2021, UK-trained candidates had pass rates of up to 83%, compared with 52.0–56.7% among international medical graduates (IMGs).^1^

To address the limited regional support available in north-west England, we developed a free, trainee-led and consultant-supported CASC preparation course for core psychiatry trainees. The 8-week virtual programme, delivered via Zoom, included structured simulations and feedback from consultants and higher specialty trainees. The course design followed Kern’s six-step framework for curriculum development, ensuring it was targeted, evidence-based and sustainable.^2^

A mixed-methods evaluation was conducted using pre- and post-course surveys and a focus group, integrating quantitative and qualitative data for cross-validation. Reflexivity was maintained through regular team discussions to minimise bias, and we adhered to the Standards for Reporting Qualitative Research and Consolidated Criteria for Reporting Qualitative Research guidelines.^3^

Of the 31 trainees invited, 28 completed the course and subsequently sat the September 2022 CASC examination; 18 completed post-course evaluation surveys. Participants reported a significant improvement in confidence and exam preparedness. They highlighted the value of small-group simulations and real-time consultant feedback in developing communication skills and clinical reasoning. High costs and limited access to external commercial courses were identified as barriers to preparation, underscoring the importance of free regional initiatives.

The cohort achieved an overall 86% pass rate, including a 78.6% pass rate among IMGs, substantially exceeding national averages of 77.2% and 53.6%, respectively. These findings suggest that regionally delivered, simulation-based training may contribute to narrowing the attainment gap between UK and IMG trainees. Our model echoes outcomes from the RCPsych Masterclass, a targeted educational intervention supported by the General Medical Council and Health Education England, which reported a 12.9% improvement in IMG pass rates.^4^ The initiative also aligns with the NHS Long Term Workforce Plan, which emphasises blended and simulation-based learning across all medical specialties.^5^ Importantly, the course has proven sustainable. It has now been embedded within the local academic training programme for the past 4 years with similar high pass rates, with delivery by previous participants ensuring continuity and scalability. This pilot supports the value of trainee-led, simulation-based teaching in enhancing examination performance and promoting equity in postgraduate psychiatry training.
